# Outcomes of Intracytoplasmic Sperm Injection Cycles for Complete Teratozoospermia: A Case-Control Study Using Paired Sibling Oocytes

**DOI:** 10.1155/2015/470819

**Published:** 2015-12-29

**Authors:** Nigel Pereira, Queenie V. Neri, Jovana P. Lekovich, Steven D. Spandorfer, Gianpiero D. Palermo, Zev Rosenwaks

**Affiliations:** Ronald O. Perelman and Claudia Cohen Center for Reproductive Medicine, Weill Cornell Medical Center, 1305 York Avenue, New York, NY 10021, USA

## Abstract

*Objective*. To investigate the outcomes of intracytoplasmic sperm injection (ICSI) cycles where sibling oocytes from a single donor were split between two recipients based on strict sperm morphology.* Methods*. Retrospective cohort study. All ICSI cycles had one donor's oocytes split between two recipients in a 1 : 1 ratio based on strict sperm morphology, that is, one male partner had morphology of 0% and the other had morphology of >1%. Fertilization, positive hCG, clinical pregnancy, spontaneous miscarriage, and live birth rates of the aforementioned groups were compared.* Results*. The baseline characteristics of the two groups (*n* = 103), including semen parameters of the male partners, were comparable. There was no difference in the fertilization rates when comparing the 0% group to the >1% group (78.7% versus 81.6%; *P* = 0.66). The overall positive hCG, clinical pregnancy, spontaneous miscarriage, and live birth rates for the 0% group were 61.2%, 49.5%, 10.7%, and 38.8%, respectively. The corresponding rates in the >1% group were positive hCG (63.1%), clinical pregnancy (55.3%), spontaneous miscarriage (7.77%), and live birth (46.6%).* Conclusions*. The fertilization and pregnancy outcomes of ICSI cycles for strict sperm morphology of 0% versus morphology of >1% are equivalent. These results can provide reassurance to couples undergoing ICSI for severe teratospermia.

## 1. Introduction

Semen analysis is commonly used in the evaluation of the male partner among infertile couples [1]. Of the various semen analysis parameters, strict sperm morphology has been proposed to be the most informative in differentiating between fertile and infertile men [1]. Strict sperm morphology has been considered a biomarker of sperm fertilizing capacity, independent of motility and concentration [2]. Severe abnormalities in sperm morphology, as in the case of teratospermia, are associated with poor fertilization during in vitro fertilization (IVF) cycles [2, 3]. In such clinical scenarios, intracytoplasmic sperm injection (ICSI) results in higher fertilization rates than conventional IVF, without affecting embryo quality [4].

Previous studies have shown that sperm with poor morphology can have similar fertilization and pregnancy rates to sperm with normal morphology when ICSI is utilized [5–9]. However, there is a dearth of literature [10, 11] regarding the outcomes of ICSI cycles for teratospermia, specifically in patients with strict morphology of 0%. The primary objective of this study is to investigate the fertilization and pregnancy outcomes of ICSI cycles at our center for complete teratospermia. By using split donor oocyte cycles (sibling oocytes), we aim to isolate male factor infertility, allowing for paired-comparison of fertilization and pregnancy outcomes between couples with and without complete teratospermia.

## 2. Materials and Methods

### 2.1. Cycle Inclusion Criteria

The institutional review board at Weill Cornell Medical College approved this retrospective study protocol. All couples initiating ICSI with anonymous donor oocytes at the Ronald O. Perelman and Claudia Cohen Center for Reproductive Medicine during a 6-year period resulting in embryo transfer were analyzed for potential inclusion. Cycles utilizing conventional IVF, surgically retrieved sperm, or frozen sperm were excluded. Most often, donor oocytes are split equally between two couples at our center, provided that the total number of oocytes retrieved from the donor is ≥14. By study design, patients were assigned to two groups in a 1 : 1 fashion based on strict sperm morphology: the first group had morphology of 0%, while the second group had morphology of ≥1%.

### 2.2. Assessment of Sperm Morphology, Processing, and Sperm Injection

Semen samples were produced after 2–5 days of abstinence. Samples were evaluated for volume, total count, concentration, and motility based on WHO criteria [12]. Kruger's strict criteria were used to assess sperm morphology [3, 13]. At our center, sperm morphology is generally evaluated by 7 andrologists on a consistent basis. To minimize intraindividual and interindividual variability, our andrologists review standard sperm morphology slides on a weekly basis. Moreover, New York state mandates all andrologists to participate in proficiency testing every 6 months. Slide preparation and staining for the assessment of sperm morphology were carried out based on previously described protocols [14]. Specifically, the morphological evaluation of viable spermatozoa was carried out using an inverted light microscope (×100 lens under oil immersion) with an ocular lens (×10 magnification), allowing a total magnification of around ×1,000 [14]. Our center generally uses prestained Testsimplets (Origio, Cooper Surgical, Inc., Trumbull, CT, USA) slides to evaluate sperm morphology. In the current study, the Diff-Quick stain (Microptic S.L., Barcelona, Spain) was also used to confirm morphology results. At least 200 sperm were evaluated per slide for morphology. Any slide with a strict morphology of 0% was evaluated and confirmed by a second andrologist. Semen samples with insufficient sperm for morphologic assessment were excluded. Sperm microinjection was carried out based on previously described protocols [15, 16]. The spermatozoon selected for injection was based on head morphology, midpiece, and flagellar shape and dynamic characteristics such as swimming patterns and progression [15, 16]. Oocytes were examined 12–17 hours after ICSI for normal fertilization, that is, the presence of two distinct pronuclei (PN) and two clear polar bodies [15, 16].

### 2.3. Clinical and Laboratory Protocols

Controlled ovarian stimulation (COS), Human Chorionic Gonadotropin (hCG) trigger, oocyte retrieval, embryo culture, and ET were performed as per our standard protocols [14]. Anonymous oocyte donors were started on oral contraceptive pills (Ortho-Novum, Janssen Pharmaceuticals) for pretreatment follicular synchronization. Ovarian stimulation was carried out to maximize follicular response while minimizing the risk of ovarian hyperstimulation syndrome (OHSS). The initial gonadotropin dose was based on factors including patient age, weight, antral follicle count, and previous response to stimulation, if any. Patients were stimulated with gonadotropins followed by pituitary suppression with a GnRH antagonist (Ganirelix Acetate, 0.25 mg (Organon), or Cetrotide, 0.25 mg (EMD-Serono)) based on a previously described flexible protocol [17].

hCG was used as the ovulation trigger in the majority of cycles. Ovidrel (EMDSerono), Novarel (Ferring Pharmaceuticals), or Pregnyl (Schering-Plough) was administered according to a sliding scale (10,000 IU for E_2_ (Estradiol) <1,500 pg/mL, 5,000 IU for E_2_ 1,501–2,500 pg/mL, 4,000 IU for E_2_ 2,501–3,000 pg/mL, and 3,300 IU for E_2_ >3,001 pg/mL). Generally, the hCG trigger was given when the two lead follicles attained a mean diameter >17 mm. Oocyte retrieval was performed with transvaginal ultrasound guidance using conscious sedation approximately 35–37 hours after hCG administration. Based on our study design, each oocyte donor donated to only 2 couples: one with and one without strict sperm morphology of 0%. ICSI of donor oocytes was performed using the male partner's sperm. All embryos were cultured using in-house culture media. Recipients underwent embryo transfer (ET) on day 3 in the majority of cases. All ETs were performed with Wallace catheters (Marlow/Cooper Surgical). Luteal support in recipients with intramuscular progesterone and estradiol patches (Climara, Bayer Healthcare Pharmaceuticals) was begun on the day of retrieval.

### 2.4. Study Variables

Demographic and baseline characteristics recorded for oocyte donors included age, BMI (kg/m^2^), basal FSH (mIU/mL), basal LH (mIU/mL), and basal E_2_ (pg/mL) levels. COS parameters recorded were as follows: total days of ovarian stimulation, total antagonist days, total dosage of gonadotropins administered (IU), peak estradiol (E_2_) level (pg/mL), total number of oocytes retrieved, and total number of mature oocytes. Characteristics of recipients included age, gravidity, parity, number of oocytes fertilized, number of embryos transferred, and day of embryo transfer. For the male partner, mean age, mean spermatozoa count (×10^6^/mL), spermatozoa motility (%), and morphology (%) were also recorded. Cycle outcomes analyzed included positive hCG, clinical pregnancy, spontaneous miscarriage, and live birth rates. Clinical pregnancy rate was defined as the number of intrauterine gestations with fetal cardiac activity per cycle. Pregnancy loss after visualization of an intrauterine gestation was considered a spontaneous miscarriage. Any birth after 24 weeks of gestational age was considered a live birth.

### 2.5. Statistical Analysis

Continuous variables were checked for normality and expressed as mean ± standard deviation (SD). Nonparametric data were expressed as median (interquartile range (IQR)). Categorical variables were expressed as number of cases (*n*) and percentage of occurrence (%). Wilcoxon's rank sum test and paired* t*-test were utilized for continuous variables. Chi-square (*χ* ^2^) with Mantel-Haenszel correction was used for categorical variables. Pregnancy outcomes were controlled for donor age, hCG trigger dose, or day of ET when indicated. Statistical significance was set at *P* < 0.05. Statistical analyses were performed using STATA version 13 (College Station, TX: StataCorp LP).

## 3. Results

A total of 206 couples met inclusion criteria and underwent ICSI with donor oocytes during the study period. Of these, 103 male partners had a strict sperm morphology of 0% and ≥1%, each. The majority of recipients were Caucasian with a mean (±SD) age of 42.1 (±4.14) years. The mean age of the donors was 27.5 (±3.69) years.  Table 1 summarizes the overall demographics, baseline characteristics, and COS parameter of oocyte donors.

Table 2 compares the baseline characteristics of the study cohort. There was no difference in the mean age, gravidity, or parity of the recipients. Similarly, no difference in the mean age, spermatozoa count, and spermatozoa motility of the male partners was noted. The number of oocytes fertilized, fertilization rate, blastocyst transfer rate, and the number of embryos transferred between the two groups were comparable.

Table 3 compares the ICSI cycle outcomes of patients with and without strict morphology of 0%. The overall positive hCG, clinical pregnancy, spontaneous miscarriage, and live birth rates for the study cohort were as follows: 62.1%, 52.4%, 9.22%, and 42.7%, respectively. No difference in the positive pregnancy, clinical pregnancy, spontaneous miscarriage, and live birth rates was noted between the two groups.

## 4. Discussion

Our study shows that the fertilization and pregnancy outcomes of ICSI cycles for strict sperm morphology of 0% versus morphology of ≥1% are equivalent. Furthermore, by using sibling oocytes, the study is able to isolate and assess the effect of male factor individually.

Morphologic assessment of sperm not only is an important part of the semen analysis, but is also valuable in potentially predicting successful fertilization outcomes [11, 18]. In a systematic review of 55 publications, Ombelet et al. indicated that an inseminating motile count of >1 million/mL with intrauterine insemination (IUI) was possibly the best cost-effective treatment before starting IVF, irrespective of sperm morphology [19]. In another meta-analysis of IUI outcomes, Van Waart et al. reported a trend towards higher pregnancy rates when the strict sperm morphology was >4% [20]. The predictive value of sperm morphology on the outcomes of ICSI cycles is less clear and therefore debated [11]. Some studies have shown that sperm morphology is one of the most predictive factors for pregnancy after conventional IVF [3, 21–23]; however, several others have shown that fertilization and pregnancy outcomes of ICSI cycles for teratospermia are comparable to ICSI cycles with normal sperm morphology [5–9]. However, a majority of these studies have analyzed ICSI cycle outcomes by comparing patients with a morphology of <4% versus those with ≥4%.

Only 2 studies to date have reported the outcomes of ICSI cycles for severe teratospermia with strict morphology of 0%. In the first study, McKenzie et al. [10] reported the outcomes of 54 ICSI treatment cycles in 45 patients with strict sperm morphology of 0%. There were 21 pregnancies, with an overall pregnancy rate of 38.9% per cycle. Though a male factor control group was lacking, the authors compared this pregnancy rate to patients with tubal factor (IVF only) and reported no statistical difference between the two pregnancy rates. The authors concluded that men with severe teratospermia could achieve acceptable pregnancy rates with ICSI. In the second retrospective study of 1074 ICSI cycles, French et al. [11] compared the fertilization, pregnancy, and live birth outcomes across 8 subgroups based on strict sperm morphology, that is, 0%, 1%, 2%, 3%, 4%, 5–7%, and >7%. While the overall outcomes were similar across the subgroups, the authors did observe the highest pregnancy and live birth rates in the 0% morphology subgroup, though these differences did not achieve statistical significance. Based on these results, the authors suggested that strict sperm morphology had little prognostic value in ICSI cycle outcomes.

Our overall findings are consistent with the studies by McKenzie et al. and French et al., which show equivalent outcomes of ICSI cycles for strict sperm morphology of 0% versus morphology of ≥1%. The ability of ICSI to achieve normal fertilization independent of sperm morphology can be explained by the presence of sperm-borne oocyte activating factor [24, 25]. In an interesting set of experiments that compared fertilization of oocytes after microinjection with isolated sperm heads, tails, or head and free tail, higher fertilization rates were noted after injection with sperm heads (60.3%) versus free tails (18.2%) or head and free tails (46.2%) [26]. These data indicated that a putative oocyte activating factor is present within the sperm head, which is not affected by the overall morphology of the sperm [27]. Furthermore, in mice, microinjection of the sperm head alone has been shown to be critical for oocyte activation and subsequent embryonic development [28, 29].

A major strength of this study pertains to use of sibling oocytes, which controls for male factor diagnosis. We also acknowledge limitations of our study. First, the study did not assess the progression of supernumerary embryos to the blastocyst stage for all patients. The published literature reveals contradictory results. For example, in a study of sibling oocytes that were either inseminated or injected with sperm from patients with teratospermia, Kihaile et al. [30] found fewer ICSI embryos progressing to the blastocyst stage. In contrast, Van Landuyt et al. [31] found that sibling oocytes from ICSI versus conventional insemination showed similar rates of blastocyst formation. French et al. [11] showed highest rates of blastocyst development in the 0% morphology subgroup, likely due to the low prevalence of female factors in this group. Second, the association of other semen abnormalities such as oligospermia with teratospermia could not be assessed in this study due to its study design. However, there is robust evidence from current literature indicating that ICSI is the most effective assisted reproductive technique in enabling fertilization in severe forms of male factor indications and gamete dysfunction [32, 33]. In fact, ICSI has been used successfully in patients with extremely low concentration and progressive motile spermatozoa [33]. Third, we acknowledge the possibility of intraindividual and interindividual assessment of sperm morphology as a confounding variable [34, 35]. Finally, due to the retrospective nature of our study it is uncertain whether these findings would hold true in a prospective setting.

Current evidence suggests that the fertilization and pregnancy outcomes of ICSI cycles for severe teratospermia (strict morphology of 0%) are equivalent to those with morphology of >1% to <4% and >4%. As evident from our findings, the presence of complete teratospermia does not negatively impact embryonic development or pregnancy outcomes [11]. Though some recent studies argue the use of conventional insemination over ICSI in the setting of isolated teratospermia [36], prospective data are needed to validate this management strategy, particularly in the context of severe teratospermia. Most recently, intracytoplasmic morphologically selected sperm injection (IMSI) has been proposed as an alternative to ICSI in patients with severe teratospermia or repeated implantation failure after conventional ICSI [37]; however, current evidence does not support the routine use of IMSI in clinical practice [38]. In conclusion, our results can provide reassurance to couples pursuing ICSI for complete teratospermia and reaffirm the role of ICSI as a tool to alleviate severe male factor infertility.

## Figures and Tables

**Table 1:** Baseline and ovarian stimulation characteristics of oocyte donors (*n* = 103).

Parameter	
Age (M years ± SD)	27.5 ± 3.69
BMI (M kg/m^2^ ± SD)	22.5 ± 4.61
Basal FSH (M mIU/mL ± SD)	2.43 ± 1.37
Basal LH (M mIU/mL ± SD)	2.22 ± 1.76
Basal E_2_ (M pg/mL ± SD)	33.2 ± 11.6
Total stimulation days (M ± SD)	9.81 ± 2.65
Total antagonist days (M ± SD)	4.58 ± 1.53
Total gonadotropins administered (M IU ± SD)	2487.9 ± 790.3
E_2_ level on day of trigger (M pg/mL ± SD)	2124.4 ± 833.6
Number of oocytes retrieved (M ± SD)	15.6 ± 3.79
Number of mature oocytes (M ± SD)	12.3 ± 6.56

Data are presented as mean (M) ± standard deviation (SD) and *n* (%); BMI: body mass index; FSH: follicle stimulating hormone; LH: luteinizing hormone; E_2_: estradiol.

**Table 2:** Comparison of baseline characteristics (*n* = 206).

Parameter	0% morphology	≥1% morphology	*P*
	(*n* = 103)	(*n* = 103)	
Age of recipient (M years ± SD)	42.2 ± 4.12	41.9 ± 4.17	0.60
Gravidity (M ± SD)	1.35 ± 1.29	1.14 ± 1.27	0.24
Parity (M ± SD)	0.29 ± 0.52	0.20 ± 0.40	0.17
Age of male partner (M years ± SD)	47.1 ± 4.49	47.5 ± 4.65	0.53
Spermatozoa count (M × 10^6^/mL ± SD)	38.7 ± 4.71	37.4 ± 6.78	0.11
Spermatozoa motility (%)	40.4	39.7	0.92
Spermatozoa morphology (%)	—	2 (1–3)	—
Median oocytes fertilized	6 (5–7)	6 (5–7)	>0.99
Fertilization rate (%)	78.7	81.6	0.61
Blastocyst transfer rate (%)	13 (12.6)	11 (10.7)	0.66
No. of embryos transferred (M ± SD)	2.30 ± 0.78	2.38 ± 0.74	0.45

Data are presented as mean (M) ± standard deviation (SD), median (interquartile range), and *n* (%).

**Table 3:** Comparison of ICSI cycles outcomes of study cohort (*n* = 206).

Parameter	0% morphology	≥1% morphology	*P*
	(*n* = 103)	(*n* = 103)	
Age (M years ± SD)	42.2 ± 4.12	41.9 ± 4.17	0.60
Positive hCG rate (%)	63 (61.2)	65 (63.1)	0.77
Clinical pregnancy rate (%)	51 (49.5)	57 (55.3)	0.40
Spontaneous miscarriage rate (%)	11 (10.7)	8 (7.77)	0.47
Live birth rate (%)	40 (38.8)	48 (46.6)	0.26
Multiple pregnancy (%)	7/40 (17.5)	9/48 (18.6)	0.98

Data are presented as mean (M) ± standard deviation (SD) and *n* (%).
